# Real-world experience with venetoclax-based therapy for patients with myeloid sarcoma

**DOI:** 10.1007/s12672-024-01068-z

**Published:** 2024-06-05

**Authors:** Xinyi Jian, Jie Cha, Zhijuan Lin, Siting Xie, Yueting Huang, Yun Lin, Haijun Zhao, Bing Xu, Yiming Luo

**Affiliations:** 1 The Graduate School of Fujian Medical University, Fuzhou, 350108 Fujian China; 2 The School of Clinical Medicine, Fujian Medical University, Fuzhou, 350108 Fujian China; 3 Department of Hematology, School of Medicine, The First Affiliated Hospital of Xiamen University and Institute of Hematology, Xiamen University, Xiamen, 361003 Fujian China

**Keywords:** Myeloid sarcoma, BCL-2, Venetoclax, Extramedullary disease, Treatment, Prognosis

## Abstract

**Background:**

The treatment of myeloid sarcoma (MS) is challenging and has not markedly improved patient prognosis. The introduction of venetoclax (VEN) has changed the treatment of MS, and venetoclax-based therapy has been described as very promising in several case reports.

**Methods:**

In this retrospective study, we analyzed the treatment outcomes of 14 patients with MS treated with venetoclax-based therapy at The First Affiliated Hospital of Xiamen University from January 2020 to October 2023

**Results:**

The cohort consisted of 7 (50%) women and 7 (50%) men with an average age of 37.5 years. Four patients (28.6%) had isolated MS de novo, 2 (14.2%) were diagnosed synchronously with AML, and 8 (57.2%) had isolated extramedullary relapse. The most common sites for MS in our cohort were the skin and lung, followed by the spinal canal, soft tissue, bone and kidney. Five patients were affected at more than three sites. Nine patients received VEN in combination with azacytidine, and 5 patients received VEN in combination with other agents. The median number of venetoclax therapies administered was 2 cycles (range: 1–10 cycles). A response was observed in all patients included in the study, with 8 patients (57.2%) achieving a CR and 3 patients (21.4%) achieving a PR, corresponding to an ORR (including CR and PR) of 78.6%. The median follow-up time for all patients was 13 months (range 1–44 months), and the 1 year OS for all patients was 67.7%.

**Conclusions:**

Venetoclax-based therapy shows excellent efficacy and safety in MS patients in the “real world” at a single institution, and a corresponding prospective study is needed to verify this conclusion.

## Background

Myeloid sarcoma (MS) is defined as a tumor mass composed of myeloid blasts, with or without maturation, occurring at an anatomical site other than the bone marrow and characterized by obliteration of local tissue architecture [1]. MS can occur as isolated MS without AML, synchronously with AML diagnosis and after or at the time of AML relapse [2]. MS has been reported to occur in 2.5%–9.1% of AML patients [3].MS can manifest at any age, with a median onset age of 56 years. The male–to–female ratio is 1.2:1, with slightly more males affected than females. MS can manifest in any site, leading to a diverse range of clinical presentations. The most commonly reported sites include lymph nodes, skin and soft tissue, bone, testis, gastrointestinal tract, and peritoneum [4, 5].The clinical manifestations of MS vary based on the site of occurrence, and symptom severity is influenced by the specific site and size of MS, typically presenting as mass effect or tumor infiltration leading to organ dysfunction [6]. The diagnosis of MS primarily relies on pathological biopsy and immunohistochemistry.

The prognosis of MS is dismal, with a 3 year OS of 31.3% [7, 8], and there has been no substantial improvement over the last two decades. Without treatment, the prognosis for MS is poor, with the majority of patients succumbing. within a short timeframe. The median Overall survival (OS) time is typically less than 12 months, and the 5 year survival rate ranges between 20 and 30%. Treatment strategies for MS encompass a range of modalities, including local therapy, systemic therapy, targeted therapy, and hematopoietic stem cell transplantation [9, 10]. There are few specific treatments for MS [11] and systemic therapy is usually recommended for treating this disease in patients with AML [12]. In recent years, considerable progress in the field of molecular genetics has provided new insights into MS, gradually transforming traditional chemotherapy into targeted therapies [13–16]. Venetoclax-based therapy was described as a very promising regimen in several case reports [17–20]. This therapeutic option rapidly led to a complete metabolic response without unusual toxicity. In the present work, we report the characteristics and outcomes of 14 MS patients treated with this strategy.

## Methods

### Study population

In this retrospective study, we enrolled 14 patients with myeloid sarcoma who were treated with venetoclax (VEN) at The First Affiliated Hospital of Xiamen College from January 2020 to October 2023, and the follow-up deadline was November 2023. The diagnosis of myeloid sarcoma was based on the 2016 World Health Organization (WHO) classification of hematolymphoid tumors. The exclusion criteria were as follows: patients who had taken VEN for less than two weeks. The patients' baseline information is summarized in Table 1 and VEN-based treatment regimens and optimal treatment responses are summarized in Table 2.

**Table 1:** Baseline characteristics at diagnosis

Characteristic	Patients (n = 14)
Age, median, years (range)	37.5 (14–68)
Sex, n (%)	
Female	7(50.0)
Male	7(50.0)
MS type, n (%)	
Isolated	4(28.6)
Relapse post-SCT	4(28.6)
Extramedullary relapse	4(28.6)
Synchronous	2(14.2)
MS sites, n (%)	
< 3	9(64.3)
≥ 3	5(35.7)
ELN risk classification by genetics, n (%)	
Favorable	4(28.6)
Intermediate	5(35.7)
Adverse	5(35.7)

*MS* myeloid sarcoma, *SCT* stem cell transplant

**Table 2:** VEN-based treatment regimens and optimal treatment responses in 14 MS patients

Treatment	Patients (n = 14)
VEN-based therapy, n (%)	
VEN + AZA	9(64.3)
VEN + AZA + chida/LDAC	3(21.4)
VEN + AML-like	2(14.3)
Best response, n (%)	
ORR	11(78.6)
CR	8(57.2)
PR	3(21.4)
NR	1(7.1)
PD	2(14.3)

*AZA* azacytidine, *CR* complete remission, *MS* myeloid sarcoma, *NR* nonremission, *ORR* objective response rate, *PR* partial remission, *PD* progressive disease, *VEN* venetoclax

### Treatment schedule

Patients were treated with VEN and azacytidine alone or in combination with other therapies. VEN was prescribed at a continuous dose of 100 mg daily, with some patients taking posaconazole in combination with oral suspension (5 ml, three times daily) or voriconazole tablets (200 mg, twice daily) for 14–21 days in a 28-day cycle until disease progression or other personal reasons for discontinuation. The dose of VEN was adjusted according to the plasma concentration of venetoclax. Patients with MS without bone marrow infiltration or bone marrow remission, grade 4 neutropenia or grade 4 thrombocytopenia lasting for 7 days, had their drug suspended until recovery to grade 1 or 2, and then resumed the same dose of treatment. Azacytidine (75 mg/m2) was administered subcutaneously on Days 1 to 7 of each 28-day cycle. Chidamide was administered at a dose of 20 mg twice weekly on a continuous basis. Other regimens included VEN combined with AML-like chemotherapy, VEN combined with azacitidine and chidamide (Chida), VEN combined with azacitidine and low lose cytarabine, and cytarabine (10 mg/m2) was administered subcutaneously on Days 1 to 7 of each 28 day cycle.Among the 14 MS patients, 9 patients (64.3%) were treated with VEN combined with azacitidine (AZA). VEN combined with AZA and Chida/Low-dose Arabinosylcytosine (LDAC) was performed in 3 patients (21.4%), and VEN combined with AML-like regimens was used in 2 patients (14.3%).

### Safety

AEs were classified according to the Common Terminology Criteria for Adverse Events (CTCAE V5.0). The frequency, severity, and causal relationship of AEs were analyzed by system organ class.

### Evaluation and definition

Efficacy was assessed as the overall response rate (ORR, including complete remission [CR] + partial remission [PR]) and overall survival (OS). In addition to OS, the duration of response (DOR) for patients who achieved a CR or a PR and best of response (BOR) was also evaluated.

The response of patients at the marrow and extramedullary sites was assessed separately. The marrow response was retrospectively evaluated using the 2022 European Leukemia Network (ELN) criteria [11]. Bone marrow aspirations were performed at baseline, then after cycles 1, 2, and 4, and then after that when clinically indicated. Multiparametric flow cytometry and real-time quantitative PCR were performed on bone marrow aspirates to detect minimal residual disease (MRD). For myeloid sarcoma, ultrasound, CT, MRI, and PET-CT were used after treatment according to the involved sites. The evaluation of patient response was based on the Response Evaluation Criteria in Solid Tumors (RECIST) 1.1 [21].

### Statistical analysis

Continuous variables are presented as the median and interquartile range (IQR) or range. Categorical variables are presented as frequencies and percentages. Survival curves were generated using GraphPad Prism 9. Swimmer plot created using Excel.

## Results

### Patient and disease characteristics

A total of 14 patients with MS who received the VEN-based regimen were included in this retrospective observational study; 7 (50%) were females, and 7 (50%) were males. The median age of the patients was 37.5 years (range 14–68 years). Four patients (28.6%) had MS isolated de novo, 2 (14.2%) were diagnosed synchronously with AML, and 8 (57.2%) had an isolated extramedullary relapse, of which 4 (28.6%) were diagnosed after allo-ASCT. The most common sites for MS in our cohort were the skin and lung (n = 4 each; 28.6%), followed by the spinal canal, soft tissue, bone and kidney (n = 2 each; 14.2%). More than three sites were involved in 5 patients (35.7%). Initial cytogenetic and molecular analyses were performed only on bone marrow samples, so a normal karyotype was demonstrated in 4 patients with de novo MS. Of the remaining patients, 2 had a complex karyotype, and 1 had a 10; 11 rearrangement. Leukemia risk was classified as favorable in 4 patients (28.6%), intermediate in 5 patients (35.7%) and adverse in 5 patients (35.7%) according to the risk stratification of the European Leukemia Net (ELN). The patient and disease characteristics are listed in Tables 3 and 4.

**Table 3:** Characteristics and clinical outcomes of MS patients

No	Age/sex	Type of MS	Karyotype	Gene mutation	Previous therapy	Treatment	BOR (m)	DOR (m)	Status at disease
1	26/M	SYNC	Complex	*SF1, TL1-ERG*	AML-like	VEN + aza + LDAC	PR (1)	6	dead
2	68/M	SYNC	46, XY	NA	VEN + aza	VEN + aza	NR	-	lost
3	67/F	EMR	46, XX	*CEBPA, NPM1, RAD21, DNMT3A*	VEN + aza	VEN + AML-like	CR (2)	9	alive
4	51/F	EMR	46, XX	*FLT3-ITD, NPM1, CSF3R, PAX5, WT1*	AML-like	VEN + aza + Chida	CR (2)	11	alive
5	36/F	EMR	46, XX, inv (16) (p13q22)	*CBFβ-MYH11*	Surgery	VEN + aza	CR (2)	23	alive
6	38/F	EMR	NA	*CEBPA, NRAS,WT1,IKZF1,MPL*	AML-like	VEN + aza	PR (1)	1	alive
7	41/M	RPT	Complex	*TP53*	aza	VEN + aza	PR (1)	15	dead
8	20/F	RPT	46, XX	*AML1-ETO*	VEN + aza	VEN + aza	CR (1)	26	alive
9	35/M	RPT	47, XY, + 8	*KRAS, ASXL1*	VEN + aza + Chida	VEN + aza + Chida	CR (1)	15	alive
10	14/F	RPT	48, XX, t(10;11), + 19, + 21	*MLL-AF10*	AML-like, DLI, RT	VEN + aza	CR (3)	20	alive
11	30/M	IMS	46, XY	*EZH2*	AML-like	VEN + AML-like	PD	-	dead
12	68/M	IMS	46, XY	*JAK2, PTPN11, CREBBP*	AML-like, RT	VEN + aza	PD	-	dead
13	38/M	IMS	46, XY	*WT1, DNMT3A, CEBPA, GATA2*	AML-like, RT	VEN + aza	CR (1)	25	dead
14	37/F	IMS	46, XX	NA	AML-like, SCT	VEN + aza	CR (1)	31	alive

*AML-like* acute myeloid leukemia-like regimen, *AraC* cytosine arabinoside, *aza* azacytidine, *BOR(m)* best of response(time to reach BOR), *Chida* chidamide, *CR* complete remission, *DOR* duration of response, *DLI* donor leukocyte infusion, *EMR* extramedullary relapse, *F* female *IMS* isolated MS, *MS* myeloid sarcoma, *M* male, *NR* non-remission, *PR* partial remission, *PD* progressive disease, previous therapy, after MS diagnosis and before venetoclax-based therapy, *RPT* relapse post-stem cell transplant, *RT* radiotherapy, *SCT* allogeneic stem cell transplant, *SYNC* synchronous, *VEN* venetoclax

**Table 4:** Involved sites in MS patients

No	Age/sex	Involved sites
1	26/M	Mediastinum
2	68/M	Abdominal wall and bone
3	67/F	Medial right forearm
4	51/F	Lateral right calf, pericardium, bilateral lungs, and bilateral renal cortex
5	36/F	Intramedullary masses at the C4–C6, intraorbital mass in the right eye
6	38/F	Lung
7	41/M	Paraspinal in the lower lobe of the right lung, the Th7 vertebral body and the 7th posterior rib on the right side
8	20/F	Intramedullary masses at the C6–T2
9	35/M	Abdominal wall
10	14/F	Left breast
11	30/M	Left jaw, abdominal cavity and bowel
12	68/M	Lung
13	38/M	Left retroperitoneum, left adrenal region, and left renal fossa
14	37/F	Mediastinum, right supraclavicular and bilateral cervical lymph nodes, spleen

*F* female, *MS* myeloid sarcoma, *M* male

### Treatment and outcome

**Fig. 1:**
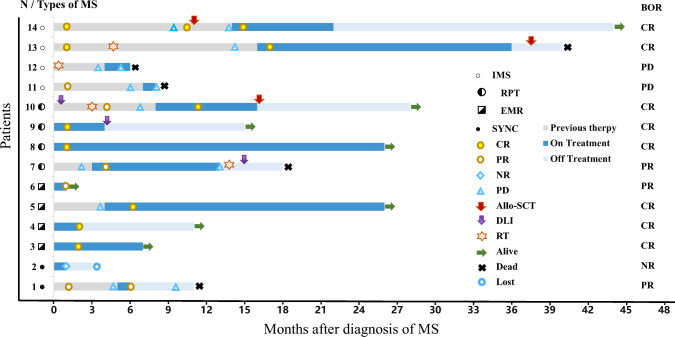
Swimmer plot of 14 patients treated with VEN-based therapies

**Fig. 2:**
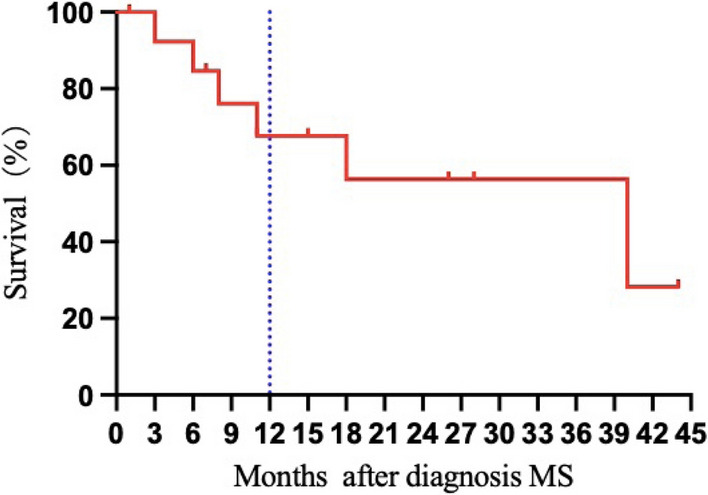
The survival probability of patients after diagnosis of MS

VEN was combined with azacytidine in 9 patients (64.3%) and with other agents in 5 patients (35.7%). The median number of venetoclax agents administered was 2 cycles (range: 1–10 cycles). All patients included in the study were evaluable for response; 8 patients achieved CR (57.2%), and 3 patients achieved PR (21.4%), corresponding to an ORR of 78.6%. Of the 8 patients who achieved CR, 7 (No.3,4,5,8,9,10,14) are in sustained remission, with a median DOR of 20 months (range: 9–31 months), the remaining one (No. 13) died of COVID-19 infection after allogeneic stem cell transplant (allo-SCT). Patient 8 was a patient who experienced extramedullary relapse after transplantation. The site of extramedullary relapse was the C6-T2 spine, and the clinical manifestation was sudden paraplegia. Patient 8 experienced rapid relief after one cycle of VEN-based therapy. Patient No. 5 had a similar experience. Both are currently receiving continuous VEN in combination with AZA, without evidence of newly developed extramedullary disease (EMD) or hematologic relapse. Patient No. 4 discontinued treatment for financial reasons, although she had a favorable response. Five of the eight patients who achieved CR did not receive additional therapy, two (No. 10, 13) underwent subsequent allo-SCT consolidation, and one (No. 9) underwent subsequent donor leukocyte infusion (DLI). Two patients (No. 1 and 7) evaluated as having a PR had complex karyotypes and poor prognoses. One refractory patient (No. 1) achieved PR after the first cycle of the VEN combination regimen. Due to irregular VEN use and not being hospitalized within the prescribed time, he experienced disease progression and died 3 months later. Patient No. 7 achieved PR after two cycles of the VEN combination regimen. Due to the progression of the extramedullary lesions, he was successively treated with radiotherapy (3000 cGy/10 f /300 cGy), chemotherapy and DLI and eventually died from infection and disease progression. Patient No. 6 was diagnosed with MS shortly before the end of the follow-up period, and only the first cycle of VEN in combination with AZA was administered before the end of the follow-up period. One month later, her lung lesions had substantially reduced, and the response was provisionally assessed as PR. She continues to be treated with VEN in combination with AZA and is planning to receive a bone marrow transplant after achieving CR. Two patients (No. 11, 12) were treated with VEN-based therapy at the stage of disease progression and died shortly after one cycle of VEN-based therapy. Patient No. 2 achieved hematologic recovery, and his abdominal wall mass shrank only 2 weeks after treatment. He was then transferred to a local hospital for treatment and was lost to follow-up. Patient 2 responded well to VEN treatment. However, he was ultimately classified as NR because the extent of improvement did not meet the criteria for PR. Figure 1 shows the outcomes of 14 patients treated with VEN-based therapies since the initial diagnosis of MS. The median follow-up duration was 13 months (range 1–44 months), and the 1 year OS for all patients was 67.7%. Figure 2 shows the survival probability of patients after diagnosis of MS.

### Safety

All patients were included in the safety assessment. The most common nonhematologic AEs were nausea (40.0%) and vomiting (20.0%). Regarding hematological toxicity, neutropenic infections were the most common and were observed in 6 patients; these infections occurred mainly in the first and second cycles of treatment.

## Discussion

In this report, we have presented preliminary evidence of the excellent efficacy and safety of VEN-based treatment in MS patients in the “real world” at a single institution. Although we are aware of potential patient selection bias for retrospective analysis, our study showed superior response rates with this strategy, with an ORR of 78.6% and a 1 year OS of 67.7% in the entire cohort, even in patients with unfavorable molecular status or multiple affected sites. According to previous studies, the median survival times of patients with isolated MS, synchronous AML and recurrent MS treated with conventional agents were only 2, 10 and 16 months, respectively [22]. Importantly, the strategy was well tolerated without excessive toxicity, making it a viable option even for frail patients.

Like our study, small retrospective studies have shown promising results in the use of VEN-based therapies in various settings [17, 23–25]. In a cohort study of 18 patients with evidence of MS, 5 with newly diagnosed AML and 13 with concurrent R/R AML, an ORR of 60% was reported for newly diagnosed AML, but only 38% was reported for R/R AML [25]. In the case of a patient with AML who experienced extramedullary multifocal recurrence after SCT, neither local irradiation nor systemic treatment with the novel CD33-targeted antibody–drug conjugate had an effect on CR, which persisted for a long time after administration of VEN [17]; these findings are similar to those of some patients in our study.

However, there are inconsistent conclusions. A retrospective series evaluating the effect of VEN-based therapy in 11 heavily treated R/R AML patients with extramedullary relapse concluded that VEN therapy had little effect on the control of EMD [26]. The author speculated that repeated exposure to traditional cytotoxic drugs could induce drug resistance or clonal evolution, compromising treatment efficacy. A French retrospective multicenter observational study revealed that more than one line of treatment increased the risk of death in patients with MS [27]. Furthermore, whether a CR can be achieved determines the patient's prognosis [28]. Considering that intensive chemotherapy has an unsatisfactory effect on the survival of patients with MS [29], our study has strengthened the opinion that the earlier use of novel agents could be beneficial for patients with MS.

Although comprehensive molecular analysis may improve prognostic assessment and influence the choice of treatment, no clear association between cytogenetics and the occurrence of extramedullary disease has been demonstrated, and the importance of molecular mutation-based treatment for patients with MS has been extensive. In our report, because no cytogenetic or molecular data on MS tumors were available, although they reflect the real-life treatment of these patients, it was difficult to determine who would benefit most from the scheme.

Regarding the influence of tumor location on patient prognosis, numerous studies have identified primary tumor location as an independent prognostic factor, e.g., the bone and central nervous system [22]. It seems that this conclusion was inappropriate for our research, as some patients with bone involvement achieved noteworthy outcomes. In addition, VEN can cross the blood–brain barrier and play a role in the treatment of leptomeningeal relapse in patients with AML [19].

This study has numerous limitations, similar to those found in other studies based on retrospective datasets. One of the most important points is that the small sample size was a major weakness that made it difficult to derive a solid conclusion. Nevertheless, this study encouraged the conclusion that a corresponding prospective study should be conducted.

## Conclusions

Venetoclax-based therapy shows excellent efficacy and safety in MS patients in the “real world” at a single institution, encouraging a corresponding prospective study to verify the conclusion.

## Data Availability

The original contributions presented in the study are included in the article/supplementary material, further inquiries can be directed to the corresponding authors.

## Abbreviations

AML: Acute myeloid leukemia
Aza: Azacytidine
AraC: Cytosine arabinoside
AEs: Adverse events
allo-SCT: Allogeneic-stem cell transplant
BOR: Best of response
CR: Complete remission
Chida: Chidamide
DOR: Duration of response
DLI: Donor leukocyte infusion
ELN: European leukemia network
EMR: Extramedullary relapse
EMD: Extramedullary disease
F: Female
IQR: Interquartile range
IMS: Isolated MS
M: Male
MS: Myeloid sarcoma
MRD: Minimal residual disease
NR: Nonremission
ORR: Objective response rate
OS: Overall survival
PR: Partial remission
PD: Progressive disease
RT: Radiotherapy
RPT: Relapse post-stem cell transplant
SYNC: Synchronous
SCT: Stem cell transplant
VEN: Venetoclax
WHO: World Health Organization
